# Contribution of extrahepatic small cells resembling small hepatocyte-like progenitor cells to liver mass maintenance in transplantation model of retrorsine-pretreated liver

**DOI:** 10.1186/2193-1801-2-446

**Published:** 2013-09-08

**Authors:** Hiromichi Maeda, Yoshihiro Ota, Yongchung Wang, Kalyani Ramachandran, Robert A Montgomery, George Melville Williams, Zhaoli Sun

**Affiliations:** Department of Surgery, Johns Hopkins University School of Medicine, 720 Rutland Avenue, Ross Research 771, Baltimore, MD 21205 USA; Department of Surgery and Cancer Treatment Center, Kochi Medical School, Kohasu Oko-cho, Nankoku-city, Kochi, 783-8505 Japan; Department of Surgery, Tokyo Medical University, Tokyo, Japan

**Keywords:** SHPC, Liver transplantation, Retrorsine, GFP

## Abstract

**Purpose:**

Retrorsine selectively inhibits hepatocyte proliferation and following liver injury evokes small hepatocyte-like progenitor cells. The aim of this study is to find out whether endogenous extrahepatic cells contribute to small hepatocyte-like progenitor cells after retrorsine treatment.

**Methods:**

Wild-type Lewis rat liver exposed to retrorsine was transplanted into GFP transgenic Lewis rat. GFP positive, albumin-producing polygonal cells were expected as reciepient-derived hepatocyte-like cells.

**Results:**

Four weeks after transplantation of 50% volume of retrorsine-pretreated liver, the rate of GFP positive hepatocyte-like cells was 0.02365%. Majority of these cells resided as single cells and their cell size was significantly larger than that of normal hepatocytes (mean cell size; 799.4 um^2^ vs. 451.3 um^2^, *p*<0.0001). At eight weeks, clusters of GFP positive small-size albumin-producing cells appeared and occupied 0.00759% of hepatocytes. The morphology of these cells was similar to that of small hepatocyte-like progenitor cells, 12.5% of them were Ki67 positive, majority of them were negative for CYP1A2 staining, and some clusters contained larger cells indicating further maturation.

**Conclusion:**

Endogenous extrahepatic cells can form a cluster of small cells resembling small hepatocyte-like progenitor cells in a transplanted retrorsine-pretreated liver. The contribution of extrahepatic cells to liver mass maintenance is quite low and its importance is unclear.

**Electronic supplementary material:**

The online version of this article (doi:10.1186/2193-1801-2-446) contains supplementary material, which is available to authorized users.

## Introduction

Under normal condition, liver regeneration is completed through mitosis of mature hepatocytes. When proliferation of hepatocytes is selectively inhibited by retrorsine treatment, albumin-producing small cells termed small hepatocyte-like progenitor cells (SHPCs) arise in response to liver injuries (Gordon et al. 2000a, b Vig et al. 2006 Serra et al. 2012). SHPCs form a nodule without a capsule as a result of expansive proliferation, and restore the liver mass. In terms of the cellular origin of SHPCs, several studies did not conclusively determine whether SHPCs were originated from hepatic oval cells (Vig et al. 2006 Chen et al. 2013) or mature/maturing hepatocytes (Gordon et al. 2000a, b Ichinohe et al. 2012). However, the involvement of bone marrow cells to the formation of nodule of SHPCs is considered less likely (Vig et al. 2006).

In order to study the contribution of bone marrow (BM) stem cells to hepatocytes, BM transplantation models have been often used. However, there is a criticism that procedure-related cell injuries might alter the kinetics of stem cells. In the current study, therefore, we used liver transplantation (LT) model (Tomiyama et al. 2007) instead of bone marrow transplantation model to study the contribution of extrahepatic cells to hepatocyte in a retrorsine pretreated liver. As a result, a cluster of small cells closely resembling SHPCs was identified in the retrorsine-pretreated liver.

## Methods

### Animals

Green fluorescence protein (GFP) transgenic Lewis rats were obtained from the national Institute of health (NIH)-funded Rat Resource and Research Center, University of Missouri, Columbia, MO. Male wild type Lewis rats were purchased from Harlan Sprague-Dawley (Indianapolis, IN). Animals were maintained in the specific pathogen-free facility of the Johns Hopkins Medical Institutions and were cared for according to NIH guidelines and under a protocol approved by the Johns Hopkins University Animal Care Committee. Male rats weighting 200-225 g (approximately 8-10 weeks old) received intraperitoneal administration of retrorsine. Male rats weighting 230-280 g were used for liver transplantation.

### Liver transplantation (LT)

In order to minimize variation, H. Maeda performed LT exclusively. LT was performed using cuff technique with minor modifications (Kamada and Calne 1979 Tomiyama et al. 2007). A donor liver was perfused with cold saline containing 20 units of heparin. For transplantation of a reduced size (50%) liver, left lobe, the left side of right lobe and caudate lobe were resected immediately before saline perfusion. The duration of anhepatic phase, from clamp of portal vein to unclamping of infrahepatic inferior vena cava, was between 15 and 19 minutes. After surgery, all rats were kept individually for at least one month. In the case of death or severe jaundice judged by the color of urine and palm, the rats were excluded from the experiment and additional transplantations were performed to replenish the lost. Severe jaundice was mainly caused by bilestone in common bile duct.

### Experimental design and retrorsine pretreatment

**Figure 1:**
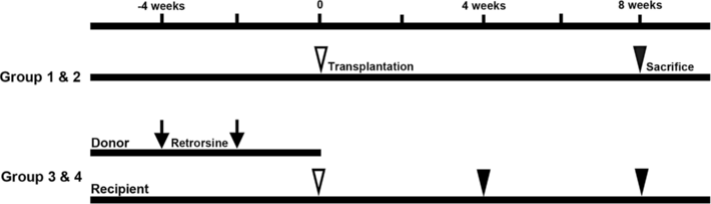
**Time line of the experiment.** Group 1 and 2 received whole liver or reduced size (50%) LT (white arrow head) and were sacrificed at 8 weeks (black arrow head). In Group 3 and 4, the donor rats received 35 mg/kg of retrorsine (black arrow) twice two weeks apart. Group 3 received whole LT, while Group 4 received 50% LT. Half of them (n=3) were sacrificed at four weeks and rest of them were sacrificed at eight weeks.

LT was performed under four different conditions (Figure 1): Group 1, a whole liver from wild-type Lewis rat was transplanted into GFP transgenic Lewis recipient; Group 2, a reduced size (50%) liver from wild-type Lewis rat was transplanted into GFP transgenic Lewis recipient; Group 3, a whole liver from retrorsine pretreated wild-type Lewis rat was transplanted into GFP transgeneic Lewis recipient; Group 4, a reduced size liver (50%) from retrorsine pretreated wild-type Lewis rat was transplanted into GFP transgenic Lewis recipient. Donor rats of Group 3 and 4 received 35 mg/kg of retrorsine (Sigma-Aldrich, St. Louis, MO) intraperitonially twice two weeks apart. Retorsine pretreated liver was harvested two weeks after the second retrorsine injection. Animals were sacrificed at four or eight weeks after transplantation.

### Definition of hepatocyte-like cell of extrahepatic cell origin

A hepatocyte-like cell derived from extrahepatic sources was defined as a large polygonal cell which resides in a hepatic cord, expresses GFP in both nucleus and cytoplasm identical to a hepatocyte in GFP transgenic rat (Additional file 1: Figure S1) and exhibits positive staining with anti-albumin antibody (Tomiyama et al. 2007).

### Sample preparation and calculation of the contribution rate

One or two month after transplantation, the rats were sacrificed by exsanguination under general anesthesia. The liver was slowly perfused with 50 ml PBS via portal vein, and followed by 50 ml 2% paraformalin. Excised liver was cut into small pieces (5-10 cubic mm) and further fixed with 2% paraformalin for one hour at room temperature in dark. Liver tissues were then incubated in 30% sucrose overnight at four degrees. Two to four pieces of liver samples were randomly embedded in O.C.T. Compound (Sakura Finetek USA, Torrance, CA) and stored in a -80 degree freezer.

To quantitatively analyze the recipient-derived hepatocyte-like cells, pictures of the tissue sections were taken with reference (1 square cm) and then areas of the sections were measured by using Adobe Photoshop CS3 (Adobe systems, San Jose, CA). Then, the number of hepatocytes within 0.16 mm^2^ from 10 microscopic random fields was examined. Subsequently, 144 cells in the liver without retrorsine treatment and 109 cells in retrorsine-pretreated liver were counted on average. After screening the whole specimen, the rate of extrahepatic cell contribution was calculated. In order to avoid counting the same cell redundantly, the sections were prepared at least 36 μm apart from each other.

### Immunoflourescence

Six μm tissue sections were incubated with 1% SDS (detergent) in PBS for 12 minutes. For albumin staining, sections were incubated with sheep anti-rat albumin antibody (1:800 dilution, BETHYL laboratory Inc, Montgomery, TX) for 30 min, followed by Cy3-conjugated donkey anti-sheep IgG antibody (1:400 dilution, Jackson immunoreserch laboratories, west Grove, PA) for 30 min. For Ki67 staining, sections were incubated with mouse anti-human Ki67 antibody (1:50 dilution, BD Pharmingen, San Jose, CA) for 60 min, followed by Cy3-conjugated donkey anti-mouse IgG antibody (1:100 dilution, Jackson immunoreserch laboratories) for 30 min. For CYP1A2 staining, sections were incubated with mouse anti-rat CYP1A2 (1:100 dilution, Abcam, Cambridge, MA) antibody for 60 min, followed by Cy3-conjugated donkey anti-mouse IgG antibody for 30 min. Nuclear staining was performed with DAPI (DAKO, Cambridgeshire, UK) when necessary. Serum blocking was performed with 10% donkey serum.

### Immunohistochemistry

The tissue sections were incubated with 1% SDS in PBS for 12 minutes. Then, they were incubated in 1% H_2_O_2_ in PBST for 25 min. After avidin, biotin block (Avidin blocking system; DAKO), sections were incubation with biotin conjugated anti-GFP antibody (Abcam) for one hour. Tissue sections were then incubated with AB complex (VECTASTAIN ABC kit; Vector, Burlingame, CA) for 30 min according to the manufacture instruction and reacted with DAB (Liquid DAB+ Substrate Chromogen System; DAKO). Counterstaining was performed by using Haematoxylin for 20 seconds. After each step, sections were adequately rinsed with PBST. When the same specimen was stained for GFP following albumin observation, incubation with 1% SDS was not performed again.

To evaluate proliferation of oval cells after LT, OV-6 expression was studied on immunohistochemistry staining. Sections were incubated in 1% H_2_O_2_ in PBST for 25 min. Then, non-specific staining was reduced by incubation with 5% donkey serum for 10 min. After avidin biotin block, sections were incubated with anti-OV-6 antibody (1:50 dilution, Santa Cruz Biotechnology, Dallas, TX) for one hour followed by biotin-conjugated anti-mouse antibody (1:100 dilution, Jackson immuneresearch laboratories) for 30 min. The signal was amplified with AB complex and detected with DAB.

### Statistical analysis

Statistical analysis and graphic depiction was performed by using KaleidaGraph version 4.0 (synergy software, Reading, PA) or Microsoft Office Excel (Microsoft, Redmond, WA). A p- value of less than 0.05 was considered statistically significant. The method of analysis is described individually.

## Results

### Mortality and exclusion of rats

Most recipients survived (19 out of 20, 95%) irrespective of whether rats received retorsine-pretreated or untreated livers. One recipient rat died and one rat was excluded due to severe jaundice. Additional LT was uneventfully performed.

### GFP positive hepatocyte-like cells in livers without retrorsine treatment (Group 1 & 2)

**Figure 2:**
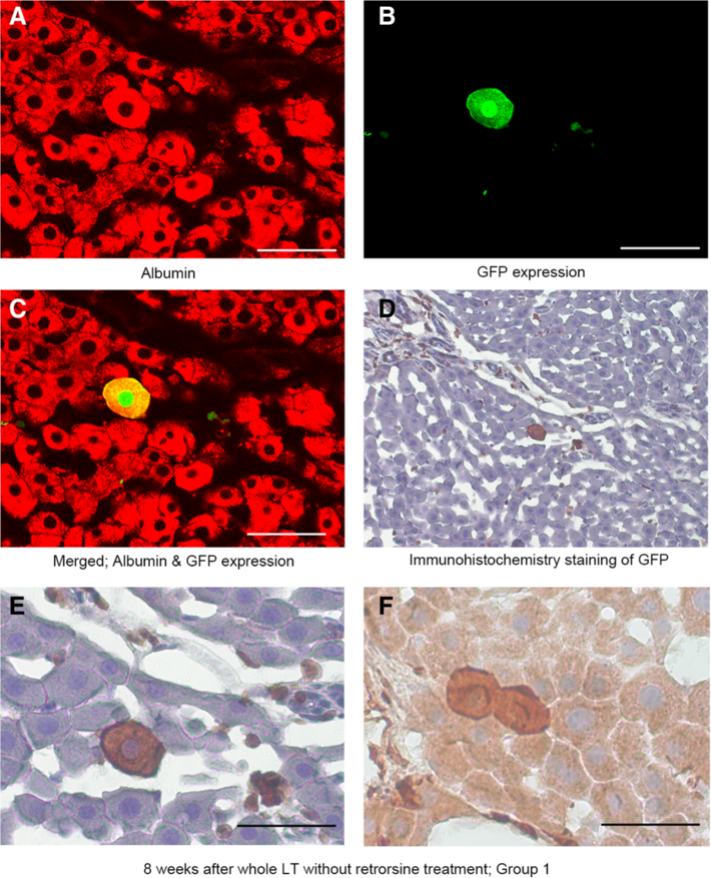
**GFP positive hepatocyte-like cells.** GFP positive hepatocyte-like cells in Group 1 is shown. **(A)** Albumin staining. **(B)** GFP expression. **(C)** Merged image. **(D)** IHC staining for GFP. **(E)** Higher magnification of **D**. **(F)** Double cells. Bar is 50 um.

For each individual, eight to 12 separate sections were examined. GFP positive hepatocyte-like cells contribute to approximately 0.00186% of total hepatocytes at eight weeks after whole liver transplantation (Group 1) (Table 1 and Figure 2A-E). Roughly, if 1 cm^2^ of specimen is screened, one or two GFP positive hepatocyte-like cells are found. The localization of these cells seems to be haphazard and neglecting the acinar zone. Among 56 randomly examined GFP positive hepatocyte-like cells, 10 hepatocytes (17.9%) were located adjacent to each other (Figure 2F). Reduced sized liver transplantation (Group 2) did not augment the contribution rate of GFP positive hepatocyte-like cells. Neither nodule of SHPCs nor clusters of GFP positive cells were identified.

**Table 1:** **Rate of extrahepatic cell contribution to hepatocytes after retrorsine-pretreated liver transplantation**

	Screened hepatocytes (cells)	GFP positive hepatocyte-like cells (%)	Cluster of cells(%)
**Group 1 (Whole LT) 8 weeks**			
No.1	1326229	0.00203	-
No.2	702764	0.00192	-
No.3	1229473	0.00154	-
Average		0.00186	-
**Group 2 (50% LT) 8 weeks**			
No.1	941860	0.00180	-
No.2	1247150	0.00208	-
No.3	2397670	0.00125	-
Average		0.00171	-
**Group 3 (whole LT& retrorsine) 4 weeks**			
No.1	741873	0.00471	-
No.2	640212	0.02218	-
No.3	1081604	0.00320	-
Average		0.01004	-
**Group 3 (whole LT &retrorsine) 8 weeks**			
No.1	67241	0.04462	0.00357
No.2	400033	0.00475	0.00650
No.3	495994	0.00323	0.01270
average		0.01753	0.00759
**Group 4 (50% LT& retrorsine) 4 weeks**			
No.1	404164	0.02944	-
No.2	311261	0.01799	-
No.3	603841	0.02351	-
Average		0.02365	
**Group 4 (50% LT& retrorsine) 8 weeks**			
No.1	529228	0.00747	0.00624
No.2	438945	0.01618	-
No.3	797411	0.00778	0.00278
Average		0.01056	0.00300

### Histological changes after transplantation of retrorsine-pretreated liver

**Figure 3:**
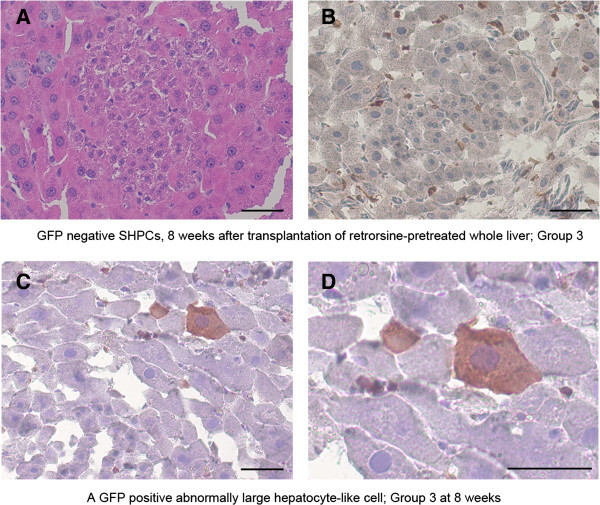
**Pathological features of retrorsine-pretreated liver after LT and GFP positive hepatocyte-like cells. (A)** and **(B)** Eight weeks after whole transplantation of retrorsine-pretreated liver shows nodules of SHPCs. **(A)** Hematoxylin Eosin staining after 10% formalin fixation. **(B)** GFP staining and Hematoxylin staining after 2% paraformalin fixation. The cell structure is well preserved. The surrounding hepatocytes are larger than normal. **(C)** and **(D)** Four weeks after transplantation of 50% retrorsine pretreated liver shows abnormally large hepatocytes and SHPCs. **(C)** IHC staining for GFP. Observation of serial section confirmed the fragment of GFP positive cell is a part of the main cell. Lower part of the picture is SHPCs. **(D)** Higher magnification of **(C)**. Bar is 50 um.

A few small nodules of SHPCs and a few abnormally enlarged hepatocytes (megalocytes) were observed four weeks after retrorsine-pretreated whole liver transplantion (Group 3). However, the number of nodules of SHPCs increased and larger nodules of SHPCs were observed at eight weeks after transplantation (Figure 3A&B and Additional file 1: Figure S2). In contrast, the SHPCs and megalocytes were abundant in reduced sized retrorsine treated livers (Group 4) at four weeks after transplantation. These findings resembled histological changes in the liver exposed to retrorsine-pretreatment and partial hepatectomy (Gordon et al. 2000a, b). The nodules of SHPCs replaced majority of the liver tissues and megalocytes were observed less frequently at eight weeks in Group 4.

### GFP positive hepatocyte-like cells and cluster of small cells in livers with retrorsine-pretreatment (Group 3 & 4)

**Figure 4:**
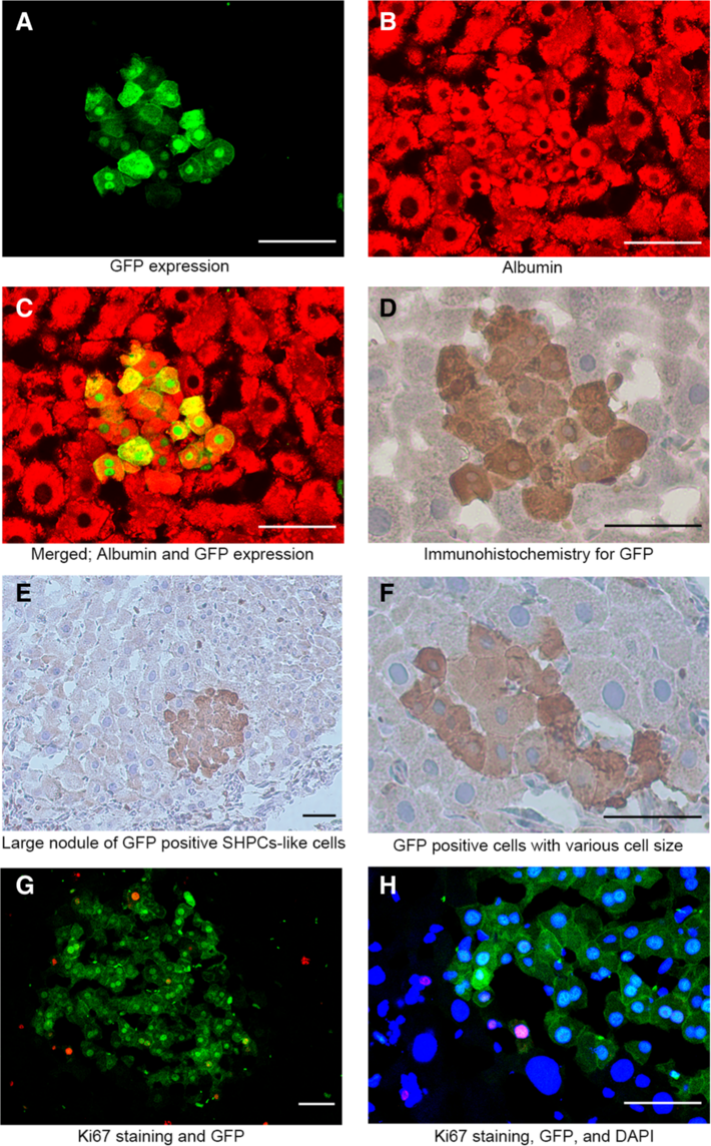
**Clusters of GFP positive SHPCs-like cells in retrorsine-pretreated liver.** Eight weeks after transplantation of retrorsine-pretreated liver, clusters of GFP positive cells are observed. These cells were not observed in Group 1 or 2. **(A)** Albumin staining. **(B)** GFP. **(C)** Merged image. **(D)** IHC staining of GFP for the same section. Nodule contains approximately 20 GFP positive cells in this plain. Surrounding hepatocytes are slightly larger than normal size hepatocytes. **(E)** Larger size nodule contains approximately 40 GFP positive cells. The nodule is beginning to compress the surrounding hepatocytes. Right upper part is nodule of GFP negative SHPCs. Note that the magnification is lower than **D** or **F**. **(F)** A cluster containing several sizes of GFP positive cells is also observed at lower frequency. A cluster of GFP positive SHPCs-like cells shows Ki67 positive staining. **(G)** Merged image of GFP (green) and Ki67 (red). **(H)** Higher magnification of same field with nucleus staining (blue). The Cluster of cells is surrounded by megalocytic hepatocytes with larger nucleus. Bar is 50 um.

**Figure 5:**
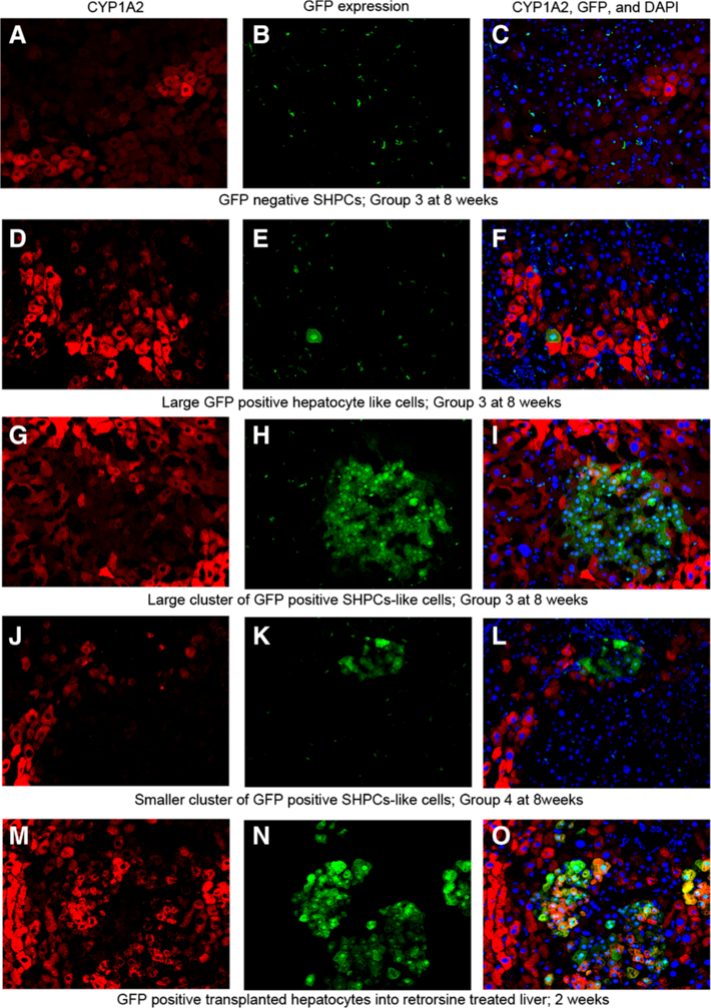
**Expression of CYP1A2 in retrorsine pretreated liver eight weeks after transplantation.** (Left column) CYP1A2. (Middle column) GFP. (Right column) Merged image of CYP1A2, GFP and nucleus (blue). **(A)**-**(C)** Nodule of SHPCs (upper side of the picture) is expansively growing and compressing surrounding hepatocytes which have CYP1A2 expression. **(D)**-**(F)** Large hepatocyte-like cells (megalocyte) are weekly positive for CYP1A2. **(G)**-**(I)** Majority of the GFP positive SHPCs-like cells lack in CYP1A2 expression. Some of them express CYP1A2 weekly. **(J)**-**(L)** Small cluster of GFP positive small cells are surrounded by CYP1A2 positive larger cells. Nodule of SHPCs is also observed (lower part of the picture). **(M)**-**(O)** 14 days after hepatocyte transplantation in retrorsine-pretreated hepatectomized liver. Formation of nodules by transplanted hepatocytes (GFP positive) and endogeneous SHPCs (GFP negative) are observed. Majorities of transplanted hepatocytes are CYP1A2 positive, opposite of GFP positive SHPCs-like cells.

In Group 3, the rate of GFP positive hepatocyte-like cells was 0.01004% at four weeks and 0.01753% at eight weeks (Table 1), and 4.5% of these cells resided adjacent to each other. Some of these cells were abnormally large and irregular in shape (Figure 3C&D). Notably, clusters of GFP positive small cells (> three cells) were observed in Group 3, which occupied 0.00759% of total hepatocytes at eight weeks after transplantation (Figure 4A-F). The size of cells and each cell’s nucleus was mostly homogenous within the cluster (Figure 4E), and it varied among clusters. Occasionally, a cluster contained different sizes of GFP positive hepatocyte-like cells within it (Figure 4F). Immunofluorescence staining demonstrated that these GFP positive small cells were albumin producing cells (Figure 4C). And some of these cells were positive for Ki67 staining (Figure 4F&G). However, the majority of them were negative for P450 (CYP1A2) and rest of them were very weakly positive (Figure 5G-L), which was contrast to transplanted hepatocytes (Additional file 1: Figure S3). Thus, these GFP positive small cells closely resemble SHPCs and we called them as GFP positive SHPCs-like cells.

The rate of GFP positive hepatocyte-like cells in retrorsine treated small livers (Group 4) was 0.02365% at four weeks. Majority of GFP positive hepatocyte-like cells were abnormally large as observed in Group 3. However, the rate GFP positive hepatocyte-like cells decreased at eight weeks (0.01056% vs. 0.02365%, *p*<0.05, Mann-Whitney U test) as the size of nodule of GFP negative SHPCs enlarged. Clusters of GFP positive cells were also identified in tissue sections at 8 weeks in Group 4, which rate was also very low (Additional file 1: Table S1).

### Size of GFP positive hepatocyte-like cells and SHPCs-like cells

**Figure 6:**
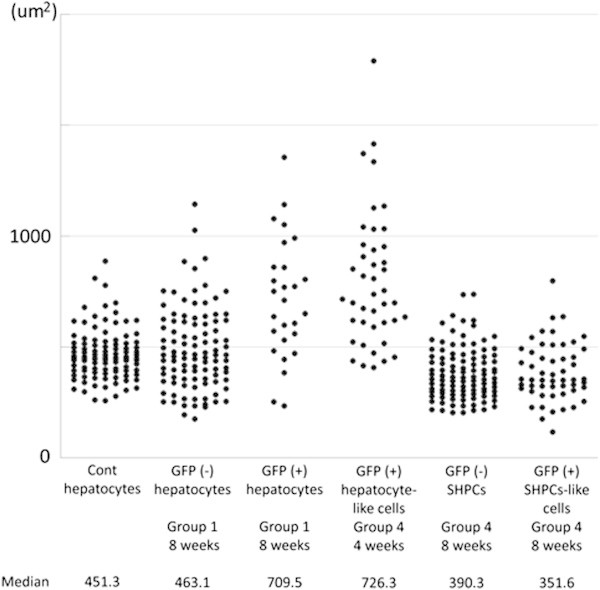
**Distribution of hepatocyte cell size.** The cell size of five different types of cells is compared. First 100 cases were presented in dot plot, if the measurement exceeded 100 cells. The size of GFP negative (-) hepatocyte in Group 1 is widely distributed and larger than that of normal liver without any treatment (Cont) (*p*=0.049). GFP positive (+) hepatocyte-like cells in Group1 and Group 4 at 4 weeks are significantly larger than those of the others (*p*<0.001). However, no significant difference was detected between GFP (+) hepatocyte-like cells from Group1 and 4 (*p*=0.457). GFP (+) SHPCs-like cells and GFP (-) SHPCs are smaller than GFP (-) hepatocytes in Group 1 (*p*<0.01). Analysis was performed by non-repeated ANOVA with post-hoc test (Scheffe) and *p*<0.05 was considered significant.

The sizes of hepatocytes were measured for quantitative analysis (Figure 6). The detail of the methods and results of statistical analysis are described in Additional file 1: Information S1 and Table S2. Briefly, cells were classified into six categories: 1, hepatocytes in normal livers (control); 2, GFP negative hepatocytes in Group 1; 3, GFP positive hepatocyte-like cells in Group 1; 4, GFP positive hepatocyte-like cells in Group 4; 5, GFP negative SHPCs in the liver graft; 6, GFP positive SHPCs-like cells. The cells size was compared by using non-repeated ANOVA and post-hoc test (Scheffe).

After transplantation, the size of GFP negative hepatocytes was widely distributed and slightly larger than that of hepatocytes in nontransplanted normal livers (median; 463.1 μm^2^ vs. 451.3 μm^2^, *p*=0.049). GFP positive hepatocyte-like cells in the livers of Group 1 and 4 were significantly larger than GFP negative hepatocytes in Group 1 and control (median; 709.5 μm^2^ and 726.3 μm^2^ versus 463.1 μm^2^ and 451.3 μm^2^, *p*<0.0001), although the some of these cells were similar to normal hepatocytes in size. The size of GFP positive SHPCs-like cells was similar to GFP negative SHPCs (351.6 and 390.3 μm^2^, respectively), and both of these cells were smaller than GFP negative hepatocytes in Group 1 (*p*<0.01).

### Proliferation of GFP positive SHPC-like cells

To find whether GFP positive SHPCs-like cells were undergoing proliferation, liver tissue sections were stained with Ki67 antibody (Additional file 1: Information S2). In normal liver (without liver transplantation), approximately 0.85% of hepatocytes were positive for Ki67 (Table 2). There was no difference in Ki67 expression between GFP negative hepatocytes and GFP positive hepatocyte-like cells in Group 1 (4.70% vs. 4.55%, *p*=0.81, Pearson’s chi-square test). Notably, the rate of Ki67 positive cells in small nodules of GFP negative SHPCs (less than 50 cells on examined pane) was high (9.32%) while the rate was low (4.11%) in larger nodules (more than 100 hepatocytes on examined plane), probably reflecting the maturation of composing hepatocytes. GFP positive SHPC-like cells showed high rate of Ki67 positive cells (12.5%).

**Table 2:** **Rate of Ki67 positive hepatocyte**

	Controlhepatocytes	GFP (-)hepatocytes	GFP(+)hepatocyte-like cells	GFP (+)hepatocyte-like cells	GFP (-) SHPCs	GFP (+)SHPCs-like cells
**Group**	Control	Group 1	Group 1	Group 4	Group 4	Group 4
**Post LT**	-	8 weeks	8 weeks	4 weeks	8 weeks	8 weeks
**Screened cells**	1315	872	66	225	118	112
**Positive cells**	11	41	3	0	11	14
**Rate (%)**	0.84	4.70	4.55	0	9.32	12.5

### Oval cell proliferation after LT

**Figure 7:**
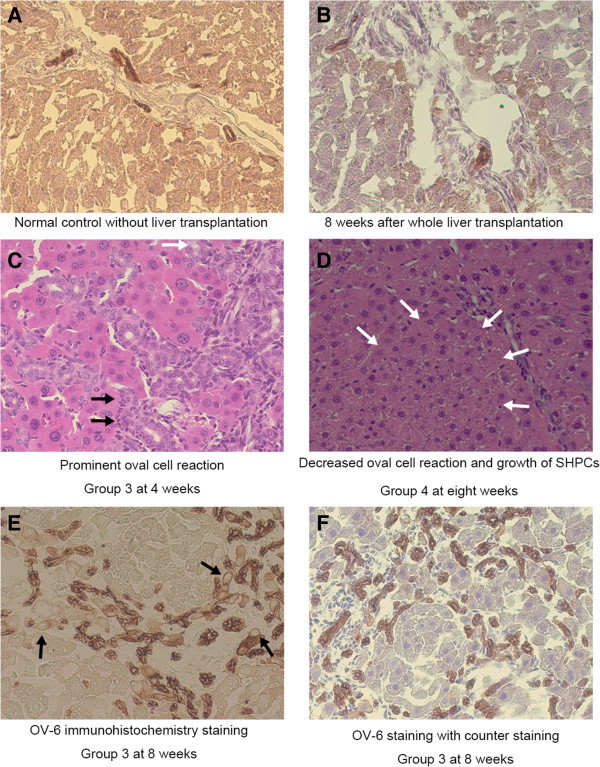
**Evaluation of ductular reaction after LT. (A)**, **(B)**, **(E)**&**(F)** OV-6 immunohistochemistry staining. **(C)**&**(D)** H.E. staining. **(A)** Normal control without liver transplantation. Only cholangiocytes are positive for OV-6 staining. **(B)** Eight weeks after liver transplantation without retrorsine treatment (Group 1), oval cell reaction is not observed. **(C)** Retrorsine-pretreated liver four weeks after whole transplantation (Group 3). The liver section shows vigorous proliferation of bile ducts and small cells with oval nucleus (black arrow). Small hepatocyte-like cells (white arrow) are scarcely observed and formation of nodules is not clear in this field. **(D)** Retrorsine-pretreated liver eight weeks after partial liver transplantation (Group 4). Nodules of SHPCs (margined with white arrow) or relatively mature hepatocytes are large and abundant. The portal area is similar to Group 1 or 2, suggesting the completion of live regeneration. **(E)**&**(F)** Eight weeks after whole transplantation of retrorsine-pretreated liver (Group 3). The oval cell reaction persists at least until eight weeks when the whole liver with retrorsine-pretreatment is transplanted. Both intermediate hepatic cells (black arrow) and nodules of SHPCs are observed.

Eight weeks after transplantation of the liver without retrorsine-pretreatment (Group 1 or 2), the periportal area showed a small amount of OV-6 positive cholangiocytes and OV-6 negative infiltrating cells (Figure 7B). Four weeks after LT of 50% volume of retrorsine-pretreated liver (Group 4), the specimen showed strong ductular reaction (Figure 7C), which subsided by eight weeks (Figure 7D). Contrarily, the ductular reaction and emergence of intermediate hepatobiliary cells persisted at least eight weeks after whole LT of retrorsine-pretreated liver (Group 3) (Figure 7E&F).

## Discussion

Hepatic injury causes occurrence of SHPCs in retrorsine-treated liver, and they gradually restore the loss of hepatocytes or replace the injured hepatocytes (Serra et al. 2012 Gordon et al. 2000a, b Vig et al. 2006). Although SHPCs have some of phenotype makers in common with oval cells or mature hepatocytes (Gordon et al. 2000a, b), the most intrinsic features of SHPCs are their small size, albumin production and formation of nodules. SHPCs also lack in expression of some CYP isozymes at immature stage. Because retrorsine is metabolized into active forms in hepatic microsomes and then selectively damages hepatocytes, the lack of CYP expression might be the mechanism of retrorsine resistance of SHPCs (Gordon et al. 2000b). The origin of SHPCs is largely endogenous cell population of the liver and contribution of bone marrow cells is considered less likely (Vig et al. 2006). In the current study using LT model, however, we identified the clusters of GFP positive small cells fulfilling these characteristic features of SHPCs for the first time. Also, GFP positive SHPCs-like cells at eight weeks showed high rate of Ki67 positive cells and several size of nucleus, suggesting their ability of proliferation and maturation. Besides, the retrorsine pretreated liver showed clusters of GFP positive hepatocytes at least more than six month after transplantation, suggesting long-term survival in the transplanted liver (data not shown). However, the biological importance of GFP positive SHPCs-like cells is unclear due to the very low rate of occurrence, and a further study is necessary.

Whether the contribution of extrahepatic cells to hepatic parenchymal cells is accomplished through transdifferentiation or cell fusion is also frequently debated. This could not be directly addressed due to the rarity of GFP positive hepatocyte-like cells and therefore the cell sizes were focused in the current study. As a result, the size of GFP positive hepatocyte-like cells was found larger than surrounding GFP negative hepatocytes in the liver of Group 1. Because GFP positive hepatocyte-like cells occasionally resided adjacent to each other or showed Ki67 positive staining, some of them are under the process of cell proliferation. However, the rate of Ki67 positive cells among GFP positive hepatocyte-like cells is not higher than that among GFP negative hepatocytes. Similarly, the GFP positive hepatocyte-like cells in retrorsine-pretreated liver showed abnormally large size and irregular shape. These results may imply that GFP positive hepatocyte-like cell is formed by cell fusion rather than transdifferentiation of extrahepatic cells into hepatocytes. Furthermore, we also performed LT transplantation of opposite direction, from GFP transgenic rat liver to a wild type rat. In this model, no GFP negative megalocytic hepatocyte was found at four weeks (Additional file 1: Figure S3). However, this approach didn’t provide a clue about the pathway of contribution of extrahepatic cells to SHPCs-like cells.

In this experiment, LT model was used instead of BM transplantation in order to find the contribution of extrahepatic cells to hepatocytes. We believe the influence on bone marrow is limited in LT model because damage on BM cells is short periods of congestion in rear part of the body during the clamping of IVC. This may explain why the current study showed GFP positive small cells resembling SHPCs and a previous experiment did not identified similar cell population (Vig et al. 2006). However, LT model also has unique problems. First, it cannot specify the origin of the extrahepatic cells. Being consistent with majority of published data, we found a very few GFP positive hepatocyte-like cells in wild-type livers after BM transplantation from GFP transgenic rats (data not shown). Therefore, BM is one of the most likely sources of hepatocytes in LT models, too. However, other organs possess resident stem progenitor cells, and these cells are potentially mobilized into peripheral circulation and take part in organ repair (Kolonin 2012). Compared to BM, from which BM cells are continuously released into peripheral blood, the contribution of stem cells from other organs under physiological condition should be minimal. However, balance of contribution may change under pathological condition (Kolonin 2012), which study has only just begun. Second, liver transplantation itself causes acute and chronic liver injuries. Abnormalities in bile duct system due to lack of arterial reconstruction (Hori et al. 2012) and the use of stent for bile duct reconstruction also caused intra- and/or extra hepatic cholelithiasis almost in all cases in this study. Probably, these factors caused differences in cell sizes and Ki67 expression of endogenous hepatocytes between control (no transplantation) and LT groups. Chronic liver injury due to cholelithiasis also seems to be the reason of gradual and persistent emergence of SHPCs and continuous oval cell reaction after whole transplantation of retrorsine-pretreated liver.

The limitation of our study is that GFP is the only marker used to detect the extrahepatic cells. Because of the very low rate of extrahepatic cell contribution, detection of X or Y chromosome as a marker of extrahepatic cells after sex-mismatch transplantation seems difficult. Also, due to the usage of paraformalin for better preservation of GFP signals, further characterization of GFP positive SHPCs-like cells by detecting the weaker cell markers was difficult. Despite limitations, the current study found that extrahepatic cells can form a cluster of small cells which closely resemble SHPCs in retrorsine-pretreated transplanted rat liver. The rate of contribution is quite low and further study is necessary to illuminate its biological importance.

## Electronic supplementary material

Additional file 1: Figure S1: Liver of GFP transgenic rat. **Figure S2**. The SHPCs after transplantation of retrorsine-pretreated liver. **Figure S3**. Hepatocyte transplantation after retrorsine-treatment. **Table S1**. Cluster of GFP positive cells that resemble SHPCs. **Information S1**. Measurement of cell size. **Table S2**. Non-repeated ANOVA and post-hoc test for the comparison of cell size. **Information S2**. Rate of Ki67 positive cells. (DOCX 3 MB)

## Abbreviations

SHPCs: Small hepatocyte-like progenitor cells
BM: Bone marrow
LT: Liver transplantation
GFP: Green fluorescence protein.
