# Supplementary material for: Contribution of extrahepatic small cells resembling small hepatocyte-like progenitor cells to liver mass maintenance in transplantation model of retrorsine-pretreated liver
Source: Springerplus. 2013 Sep 8;2:446. doi: 10.1186/2193-1801-2-446 (PMC3786066; doi:10.1186/2193-1801-2-446)
Supplement: Supplementary file 1 — Additional file 1: Figure S1: Liver of GFP transgenic rat. Figure S2. The SHPCs after transplantation of retrorsine-pretreated liver. Figure S3. Hepatocyte transplantation after retrorsine-treatment. Table S1. Cluster of GFP positive cells that resemble SHPCs. Information S1. Measurement of cell size. Table S2. Non-repeated ANOVA and post-hoc test for the comparison of cell size. Information S2. Rate of Ki67 positive cells. (DOCX 3 MB) [file 40064_2013_529_MOESM1_ESM.docx]

**Additional File**

| **Figure S1** | **Liver of GFP transgenic rat** | **Page 2** |
| --- | --- | --- |
| **Figure S2** | **The SHPCs after transplantation of retrorsine-pretreated liver** | **Page 3** |
| **Figure S3** | **Hepatocyte transplantation after retrorsine-treatment** | **Page 4** |
| **Table S1** | **Cluster of GFP positive cells that resemble SHPCs** | **Page 5** |
| **Information S1** | **Measurement of cell size** | **Page 6** |
| **Table S2** | **Non-repeated ANOVA and post-hoc test for the comparison of cell size** | **Page 7** |
| **Information S2** | **Rate of Ki67 positive cells** | **Page 8** |


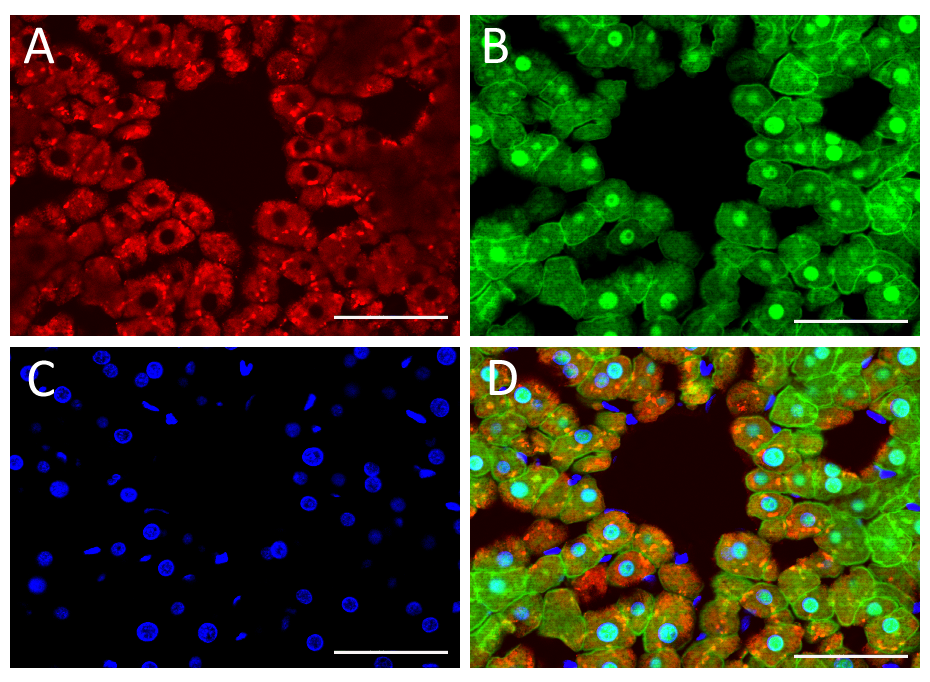


**Figure S1. Liver of GFP transgenic rat**

(A) Albumin. (B) GFP expression. (C) Nuclear. (D) Merged image. Hepatocytes of GFP positive transgenic rat have strong expression in nucleus. Non parenchymal cells of liver do not have strong GFP expression and detection of GFP of endothelial cells is difficult even in the nucleus. Bar is 50 um.


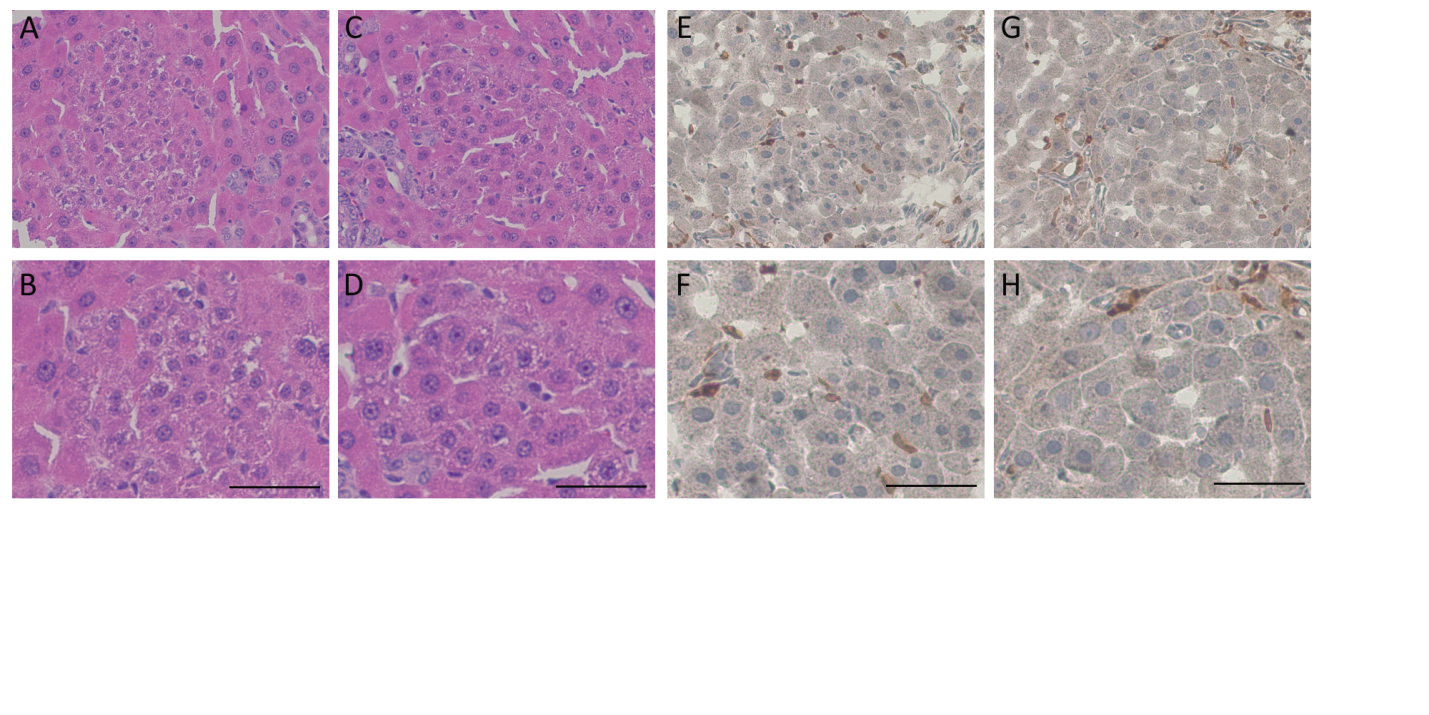


**Figure S2. The SHPCs in retrorsine-pretreated liver after LT**

A various size of nodules of SHPCs emerges in the retrorsine-pretreated liver after LT. (A)-(D) is showing Hematoxylin and Eosin staining after 10 % formalin fixation. (E)-(H) is showing hematoxylin staining after 2 % paraformalin fixation. (A) A nodule of SHPCs with smaller nucleus. (B) Higher magnification of A. (C) A nodule of SHPCs with larger cell size and nucleus. (D) Higher magnification of C. (E) A nodule of SHPCs with smaller nucleus. (F) Higher magnification of E. (G) A nodule of SHPCs which seem to have differentiated. (H) Higher magnification of G. Bar is 50 um.


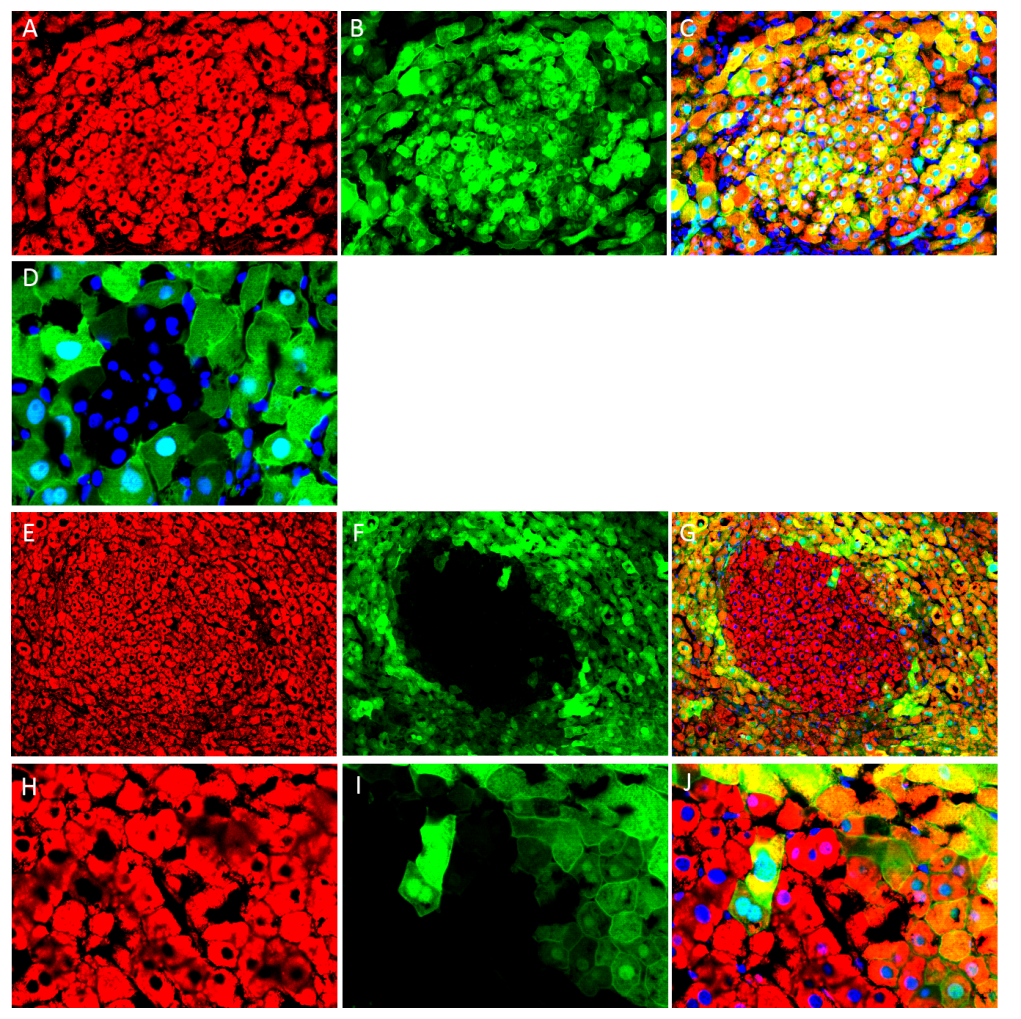


**Figure S3. LT of opposite direction and Hepatocyte transplantation after retrorsine-treatment**

(A)-(C) LT of opposite direction. (A) Albumin. (B) GFP. (C) Merged image. GFP transgenic rat was pretreated with 35mg/kg of retrorsine twice two weeks apart. Two weeks after second injection, wild-type Lewis rat received retrorsine-pretreated liver. Four weeks after LT, rats were sacrificed and sections were observed with albumin staining. No GFP negative hepatocyte-like cells is detected, which indicates that majority of contribution of extrahepatic cells might be achieved through cell fusion or stem cells are not able to differentiate into hepatocytes in GFP-rich environment. (D)-(J) Cell transplantation of wild-type Lewis hepatocytes into GFP transgenic rats after retrorsine-pretreatment and partial hepatectomy results in selective growth of transplanted hepatocytes. (D) Biopsy of liver 7 days after hepatocyte transplantation. Small clusters of hepatocytes is detected. (E)-(J) 6 weeks after hepatocyte transplantation. GFP negative hepatocytes are compressing the surrounding GFP positive hepatocytes. (E) & (H) Albumin. (F) & (I) GFP. (G) & (J) Merged images.

**Cluster of GFP positive cells that resemble SHPCs**

To determine the contribution rate, the examination for a cluster of GFP positive small cells was performed rather randomly. Therefore, sample was again screened systematically in order to elucidate how often these clusters occurred. The 6 um thickness sections of No.1 in Group 4 at eight weeks were prepared every 30 um. Totally, 20 sections were examined as shown in the Supplemental Table 1. Totally nine cluster were detected and some of them were large and found through the several sections. Total contribution rate of these cells was 0.0106 % of total number of hepatocytes.

| **Table S1. Cluster of GFP positive cells that resemble SHPCs** | | | | | | | | | |
| --- | --- | --- | --- | --- | --- | --- | --- | --- | --- |
| Slide  number | Cluster 1 | Cluster 2 | Cluster 3 | Cluster 4 | Cluster 5 | Cluster 6 | Cluster 7 | Cluster 8 | Cluster  9 |
| 1 |  |  |  |  |  |  |  |  |  |
| 2 |  |  |  |  |  |  |  |  |  |
| 3 | 6 cells |  |  |  |  |  |  |  |  |
| 4 | 13 cells | 19 cells |  |  |  |  |  |  |  |
| 5 | 10 cells | 16 cells | 9 cells |  |  |  |  |  |  |
| 6 | 9 cells | 4 cells | 7 cells | 4 cells |  |  |  |  |  |
| 7 |  |  | 4 cells |  |  |  |  |  |  |
| 8 |  |  |  |  |  |  |  |  |  |
| 9 |  |  |  |  | 7 cells |  |  |  |  |
| 10 |  |  |  |  |  | 4 cells |  |  |  |
| 11 |  |  |  |  |  |  |  |  |  |
| 12 |  |  |  |  |  |  |  |  |  |
| 13 |  |  |  |  |  |  |  |  |  |
| 14 |  |  |  |  |  |  |  |  |  |
| 15 |  |  |  |  |  |  |  |  |  |
| 16 |  |  |  |  |  |  | 4 cells |  |  |
| 17 |  |  |  |  |  |  |  |  |  |
| 18 |  |  |  |  |  |  |  | 6 cells |  |
| 19 |  |  |  |  |  |  |  |  | 11 cells |
| 20 |  |  |  |  |  |  |  |  | 17 cells |

**Information S1. Measurement of the hepatocyte cell size**

During the observation of transplanted liver under microscope, the difference of cells size was recognized. Therefore, hepatocytes and hepatocyte-like cells were devided into six groups and quantitatively compared: 1, normal hepatocytes in normal liver (Control); 2, GFP negative hepatocytes in Group 1; 3, GFP positive hepatocyte-like cells in Group 1; 4, GFP positive hepatocyte-like cells in Group 4 at four weeks; 5, GFP negative SHPCs in Group 4 at 8 weeks, 6; GFP positive SHPCs-like cells in Group 4 at 8 weeks.

For measurement of GFP negative hepatocyte in control and Group 1, pictures of randomly selected areas were taken and cell size of hepatocytes was measured in randomly chosen small areas in the picture until 500cells were counted. In order to measure the cell size of GFP positive hepatocyte like-cells in Group1, 14 separate slides were stained by GFP antibody and totally 26 cells were detected. For GFP positive hepatocyte-like cells in Group 4 at 4 weeks, 41 cells in two sections were measured. For GFP negative SHPCs, 500 cells in randomly selected nodules were measured. For GFP positive SHPCs-like cells, 52 cells from 3 representative clusters were measured.

| **Table S2. Non-repeated ANOVA for comparison of cell size** | | | | |
| --- | --- | --- | --- | --- |
|  |  | *p* value |  |  |
| Non-repeated ANOVA | | *p*<0.0001 |  |  |
| Post-hoc test (Scheffe) | |  |  |  |
|  | Comparison |  |  |  |
|  | D vs. F | *p*<0.0001 |  |  |
|  | D vs. E | *p*<0.0001 |  |  |
|  | D vs. A | *p*<0.0001 |  |  |
|  | D vs. B | *p*<0.0001 |  |  |
|  | D vs. C | *p*=0.475 |  |  |
|  | C vs. F | *p*<0.0001 |  |  |
|  | C vs. E | *p*<0.0001 |  |  |
|  | C vs. A | *p*<0.0001 |  |  |
|  | C vs. B | *p*<0.0001 |  |  |
|  | B vs. F | *p*<0.0025 |  |  |
|  | B vs. E | *p*<0.0001 |  |  |
|  | B vs. A | *p*<0.0486 |  |  |
|  | A vs. F | *p*<0.1555 |  |  |
|  | A vs. E | *p*<0.0001 |  |  |
|  | E vs. F | *p*<0.9996 |  |  |

A; Control

B; GFP negative hepatocytes in Group 1

C; GFP positive hepatocyte-like cells in Group 1

D; GFP positive hepatocyte-like cells in Group 4 at 4 weeks

E; GFP negative SHPCs in group 4 at 8 weeks

F; GFP positive small cells in cluster in Group 4 at 8 weeks

**Information S2. The rate of Ki67 positive cells**

The hepatocytes were devided into six groups as described above. For control and Group 1, pictures of 10 random magnified fields were taken and numbers of Ki67 positive and negative cells were counted. For GFP positive hepatocyte-like cells in Group 1 and 4, 25 section from Group 1 at 8 weeks and five sections from Group 4 at 4 weeks were stained, respectively. Consequently, 66 and 225 GFP positive hepatocyte-like cells were identified, respectively. For GFP negative SHPCs and GFP positive SHPCs-like cells, four nodules from Group 4 at 8 weeks were randomly chosen and examined.
